# Two Cases of Acute Poisoning from Solid Ext. Cannabis Indica

**Published:** 1890-08

**Authors:** W. B. Rushin

**Affiliations:** Calvary, Ga.


### TWO CASES OF ACUTE POISONING FROM SOLID EXT. CANNABIS INDICA.

BY W. B. RUSHIN, M. D., CALVARY, GA.

I have to report two cases of acute poisoning from solid ext. cannabis indica (McKesson and Robbins), June 20. Had under treatment for migraine, caused from functional ocular defects, Miss Lucy M., æt. 15, blonde, highly nervous temperament. She took, at 8 a. m., on morning of June 20, three-fourths grain of solid ext. cann. indica. In an hour she began to feel the characteristic effects of the drug—numbness, vertigo, visions of a delusionary character. When the eyes were shut, a feeling that she was walking on the ceiling, or head down and heels up. At 1 p. m. I was hastily summoned to her bedside. Found patient lying in bed with head and shoulders elevated. Slow, shallow and sighing respiration, flushed face, congested conjunctiva, mouth dry, pulse 126. There were no cataleptic symptoms, but loss of voluntary power to use the muscles. Involuntary muscular twitchings throughout the body ; stupor, loss of memory of past events partial, tactile sensation very much blended.

Desiring to watch the poisonous effects as to the length of time it would last and the sequelæ, etc., I withheld all treatment to counteract the physiological poisoning. I remained at the bedside of the patient for severel hours, and saw the symptoms gradually ameliorating. This patient complained of vertigo and great weakness next day. She is now taking 1-5 gr. of same preparation with the happy effect of enabling accommodation of the eyes to adjust themselves to constant work without tiring.

Case 2.—Female, white, æt. 34, steady habits, single, housekeeper. Had been suffering two weeks with persistent neuralgia in temporal branches of the fifth nerve. June 22d, gave patient three-fourths grain solid ext. cann. indica in capsule. In three hours she was drunk and hardly able to stand; flushed face and œdema, also in hands and face. She could not recollect what day of the week it was; in fact, memory was greatly impaired, and no notice was taken of the lapse of time. She fell into a stupor and slept for several hours. When awake there was experienced a feeling of impending suffocation and death. Muscular system completely relaxed; patient morose and indisposed to converse. No record was taken of the pulse or respiration.

Patient fully recovered in 24 hours without treatment.

REMARKS.—I have always used the fluid ext. heretofore of S. & D's., and have never commenced with less than 5 drops in adults, and often had to increase it to 15 before securing the characteristic effects of the drug, and then only faintly.

The results of the two doses administered in the above cases certainly illustrates in a very marked manner the wide difference in the two preparations, and the experience won has taught me the valuable lesson that all practitioners should heed in that of always beginning with the *minimum* dose of any poisonous pharmaceutical product. A suggestion that Seguin announces in his Toronto lectures to always obtain your ext. of cannabis indica from the same source should be carefully heeded.

